# How Laboratory Guidelines Promote the Validity of Circulating Extracellular Vesicle-Associated Nucleic Acid Biomarker Signatures in Liquid Biopsy

**DOI:** 10.3390/ijms262412115

**Published:** 2025-12-16

**Authors:** Michael W. Pfaffl

**Affiliations:** Animal Physiology and Immunology, School of Life Sciences Weihenstephan, Technical University of Munich, Liesel-Beckmann-Straße 1, 85354 Freising, Germany; michael.pfaffl@tum.de

**Keywords:** MIQE, MISEV, liquid biopsy, extracellular vesicles, biomarker development, validity, reproducibility, qPCR, RT-qPCR

## Abstract

Circulating nucleic acids, particularly those associated with extracellular vesicles (EVs), represent a promising class of molecular biomarkers in liquid biopsy for ‘non-invasive’ disease diagnostics, for better prognosis, and for therapeutic monitoring. However, the translation of this new circulating biomarker source into clinical practice is mostly hindered by methodological variability and a lack of standardization across the analytical workflow. This article highlights the implementation of international academic guidelines, such as Minimum Information for Publication of Quantitative Real-Time PCR Experiments (MIQE) and Minimal Information for Studies of Extracellular Vesicles (MISEV), in the entire analytical procedure in promoting the integrity, reproducibility, and validity of EV-associated nucleic acid markers in molecular diagnostics. By standardizing the liquid biopsy workflow from tissue sampling up to data analysis and statistics, these established guidelines lay the necessary scientific basis for a robust, reproducible, reliable, and valid RNA and DNA biomarker discovery in EVs. The ultimate goal is the successful implementation of the developed biomarker signature into the clinical diagnostic routine, but this requires further rounds of rigorous validation. The regularly updated guidelines should not be seen as optional recommendations, but more like an essential pillars of scientific rigor and standardization in order to achieve better and biological meaningful biomarker results in liquid biopsy.

## 1. Introduction

Recent advances in genomics and transcriptomics have revolutionized the landscape of biomarker discovery in liquid biopsy, enabling the detection of RNA and DNA molecules in body fluids such as blood, saliva, and urine. These nucleic acids, whether freely circulating, bound to proteins, or encapsulated in or attached to EVs, have demonstrated high diagnostic potential for early disease detection, prognosis, and therapeutic monitoring [1,2]. Their application in liquid biopsy holds particular promise, especially in oncology, where tumor-derived extracellular vesicles (TEV) are being intensely studied as sources of circulating nucleic acids (CNA) biomarkers that reflect tumor existence, status, and heterogeneity [2]. The inherent diagnostic value of such EV-associated nucleic acids, in particular in oncology by TEVs, has sparked widespread research efforts across academic, clinical, and industry research. Today, a growing number of experimental and clinical studies focus on the use of EVs to access disease-, tumor-, tissue-, and stage-specific molecular signatures. These include studies into various forms of circulating free RNA (cfRNA), primarily microRNAs (miRNAs) or messenger RNAs (mRNAs), as well as DNA molecules such as circulating tumor DNA (ctDNA) [3,4]. Recently, also new CNA classes, like long non-coding RNAs (lncRNAs) or miRNA isoform signatures (isomiRs) were discovered to predict therapy response in cancer patients [5], but these new classes are still in the early discovery phase. The overarching goal is the development of a robust but sensitive and early-appearing biomarker signature with a novel diagnostic value, showing high validity and reproducibility, that can be reliably detected and also quantified in clinical liquid biopsy samples [6,7].

During the last two decades, this field has received a strong boost through the application of next-generation sequencing (NGS) technologies, which offer sensitive and high-throughput detection of both known and novel RNA and DNA species [6]. Today, NGS is primarily used in biomarker discovery studies as a screening method to identify first biomarker candidates. In a second step, a smaller subset of these found biomarkers has to be validated in an independent patient cohort(s). These signatures, consisting, in general, of around 10–15 markers, are much more suitable for clinical routine diagnostics. This motivates validation of the NGS found biomarker signature by sensitive and quantitative PCR-based methods, like reverse transcription (RT) quantitative PCR (RT-qPCR) or digital PCR (RT-dPCR) [6,8]. The high resolution of these quantitative platforms has greatly expanded our understanding of the broad nature of nucleic acid EV or TEV cargos and the diversity of these signatures, associated with certain disease stages or cancer types, respectively. Among the most promising research applications and nucleic acid sources are ctDNA and cfRNA, particularly miRNAs or isomiRs, all protected or associated with EVs. In oncology, ctDNA provides valuable information about tumor mutations, methylation status, and genomic instability, while EV-associated miRNAs and RNAs offer insights into gene expression pattern, the dynamic gene regulation, and disease progression [8,9]. Therefore, the vesicular encapsulation of the CNA molecules or protection by the association to EVs, improves the nucleic acid stability by securing them from enzymatic degradation and thereby offers a direct quantification and usage as molecular biomarker. This reinforces the high potential of CNA-based biomarker signatures in molecular diagnostics as stable, robust, and valid biomarkers [6,10].

## 2. The Problem

Despite these advances, the field of liquid biopsy nucleic acid biomarker signatures faces a major obstacle—the lack of overall standardization during the biomarker development, the validation process, and the missing overall reproducibility. This consists of natural variation between different biological samples, liquid biopsy sampling, storage conditions, extraction efficiencies, contamination level, quantification and normalization methods, and many other factors in the analytical workflow, which varies between laboratories and across different studies [11].

The major EV subtypes, such as exosomes, microvesicles, and apoptotic bodies, differ in their biogenesis, size, biological markers, and molecular cargo, which influences their usefulness and robustness in biomarker discovery. These molecular and phenotypic differences enable subtype-specific isolation and molecular profiling in clinical samples [6,10]. Exosomes originate in all cell types via the endosomal pathway through intracellular sorting. They form as intraluminal vesicles within multivesicular bodies, which subsequently fuse with the outer plasma membrane. Microvesicles bud directly from the plasma membrane during cell activation, a process known as surface shedding, while apoptotic bodies arise through fragmentation and membrane vesicle formation in dying cells during programmed cell death. These different origins reflect different vesicular types and result in different molecular cargos. Exosomes transport proteins, lipids, mRNAs, and miRNAs primarily from cytoplasmic and endosomal compartments. They are often rich in tetraspanins (CD9, CD63, and CD81) as well as cytoplasmic miRNAs, which makes them stable for signal transduction over longer distances and usable as circulating biomarkers. Microvesicles are more heterogeneous than exosomes and contain a greater extent of plasma membrane components, including cell surface receptors and bioactive lipids, as well as a broader spectrum of RNA types, such as miRNA, mRNA, and lncRNA fragments. Their cargo reflects the current cellular state. Apoptotic bodies, on the other hand, contain mostly components of dying cells, larger nuclear fragments, histones, high amounts of DNA, including intact genomic DNA, mitochondrial DNA, and all RNA types, like mRNA, miRNA, ribosomal RNA, and transfer RNA [12,13]. When we aim an application in circulating biomarker detection, exosomes or small EVs are considered as very promising due to their selective cargo packaging, high stability, and molecular biomarker protection ability, especially in the context of CNA [6,8].

While the scientific community has produced an unexpectedly large amount of valuable NGS data and seminal biomarker candidates described in the scientific literature, many findings lack independent validation or cannot be translated into a reliable commercial kit and subsequently into diagnostic or clinical practice [14,15]. Basic questions remain about the analytical validity of these nucleic acid biomarkers, including whether results derived from one quantification method, normalization strategy, or given laboratory analytical workflow can be reproduced elsewhere using different methodologies, chemicals, EV purification or nucleic acid isolation kits, primer sets, or amplification and quantification platforms [16,17]. One of the core issues is the failure to confirm nucleic acid sequencing-based primarily CNA results using independent, fully quantitative methods such as (RT)-qPCR or (RT)-dPCR. Without an orthogonal sensitive and quantitative validation, it is difficult to assess the true abundance and biological relevance of the putative biomarker signature [8,16,18].

There are significant methodological variabilities between various EV purification, RNA isolation, PCR amplification and quantification approaches, and across general experimental setups. Moreover, EV purification techniques vary widely, e.g., from ultracentrifugation and size exclusion chromatography to precipitation-based or immunoaffinity-based methods, each producing different EV yields, size distributions, purity, and contamination profiles [6,19]. In addition, it was shown multiple times that RNA isolation protocols differ in their integrity and RT highly varies in their conversion efficiencies based on the RNA integrities, RT-primer selection, and selectivity for small- or long-RNA species. On the quantitative analytical side, diverse sequencing or PCR platforms, amplification methods or buffer chemistries, as well as normalization strategies are used without consensus [8,14]. Such technical variability and man-made heterogeneity summarizing over the entire quantification workflow generates unnecessary high background noise and limits inter-study or inter-laboratory comparability [15,20].

The natural occurring biological variability adds another layer of complexity. Patient heterogeneity, sample collection conditions, and patient-related factors affect the composition and abundance of EVs and the connected cfRNA and ctDNA cargo [21]. In the focus are patient factors with fluctuating or circadian characteristics, behaviors that influence health, circulating inhibitors, contaminating metabolites and individual response to treatment or applied medication. This includes intrapersonal factors like age, sex, health literacy, and, increasingly, the socioeconomic status and lifestyle, e.g., smoking, alcohol consumption, even the cultural background, as well as the ability to participate in their own healthcare.

Another challenge lies in the co-isolation of non-EV entities such as RNA-binding proteins, a huge variety of serum- or lipoproteins, and other cell-free nucleic acid families. Some EV purification methods are contaminated with unwanted abundant soluble serum proteins like albumin and globulins, various lipoproteins (e.g., HDL, LDL, VLDL, chylomicrons), and furthermore undefined large protein aggregates [11,12,13]. These (mainly) protein contaminants or inhibitors can confound the interpretation of sequencing and also the later validation of the results by PCR if not adequately removed or accounted for it. Without rigorous pre-analytical working protocols and analytical standard and/or controls, framed by international laboratory guidelines, it becomes nearly impossible to distinguish true biological results from technical artifacts or man-made variability [19,20]. The overall outcome and net effect are an erosion of confidence in the reproducibility and validity of reported biomarker signatures. Addressing these issues is essential to ensure accurate pre-analytical handling of the liquid biopsy sample, effective EV purification, pure nucleic acid isolation, and, furthermore, the precise quantification, correct normalization, and proper data analysis of the nucleic acids for a meaningful biological interpretation, leading to an ultimately reliable clinical utility of the developed biomarker signature [6,8,22].

## 3. The Solution

In response to all these challenges, the scientific community has developed over the last two decades a suite of international guidelines designed to improve the reliability of nucleic acid-based and circulating biomarker research with focus on liquid biopsy. These guidelines provide structured recommendations across the entire workflow, from sample collection and processing to data analysis and reporting, which are particularly relevant to studies involving EV-associated nucleic acids in liquid biopsy applications. Pre-analytical guidelines are essential for ensuring consistency in sample handling and processing, e.g., for blood liquid biopsy [22,23]. Variables such as EV or blood collection tubes, processing times, temperature, buffer, and storage conditions can significantly affect the quantity and integrity of nucleic acids. For plasma and serum, guidelines recommend minimizing hemolysis, centrifugation artifacts, and freeze–thaw cycles [22,24,25]. Although no single document encompasses all necessary pre-analytical factors, consensus efforts such as the SPIDIA and CEN/TS standards offer valuable recommendations for blood-derived RNA and DNA [23]. Specific initiatives for EVs are also emerging, such as EV-TRACK, which promotes transparency in reporting EV handling and isolation methods [26].

In the realm of nucleic acid quantification, the first MIQE guidelines (Minimum Information for Publication of Quantitative Real-Time PCR Experiments) were introduced in 2009 and revised quite recently in 2025 to promote methodological rigor in qPCR and RT-qPCR studies [27,28]. These guidelines emphasize the importance of assay validation, regarding RT and PCR amplification performance, primer selection and specificity, sample normalization and PCR efficiency determination strategies, just to pick some major points. The dMIQE guideline established in 2013 and the dMIQE2020 update, which extend these general MIQE principles to digital PCR, address specific parameters such as partition volume, droplet stability, and quantification thresholds [29,30]. Adherence to all these guidelines ensures that nucleic acid measurements become more reliable and consequently more biologically meaningful, based on higher analytical sensitivity, accuracy, reproducibility, and complete quantification.

Complementing these are the MISEV guidelines (Minimal Information for Studies of Extracellular Vesicles), established in 2014 and updated recently in 2024, that outline best practices for EV isolation, handling and storage of EVs, with a deep and comprehensive focus on the characterization of EVs and even functional EV studies [12,13]. It recommends using a combination of biochemical, biophysical, morphological, and imaging methods to confirm the presence, size, purity, and diverse molecular cargo of EVs (nucleic acids, proteins, lipids, and other metabolites).

With focus on nucleic acids standardization, all mentioned guidelines also extend into data analysis, bioinformatics, and statistical comparisons, which plays a critical role in high-throughput data generation, data validation and, later, reasonable biological interpretation. Algorithms for differential gene expression, sample normalization, and batch effect correction must be selected with care, and results should be reproducible with publicly available codes or workflows [6,8,18,31,32,33]. A central issue which often appears in the EV biomarker field is the inappropriate normalization of miRNAs, either for small-RNA sequencing or RT-qPCR quantification. Hence, relative differential expression studies need stably expressed miRNA reference transcripts for reliable normalization between patient or treatment groups, coming from the same type of disease or tissue type. For EV-related RNA studies, the most important experimental parameters and confounding methodological factors are the investigated tissue type, sample handling, and the performed EV or RNA isolation method. The isolated EV yield, the purified RNA amount and the resulting miRNA profiles strongly depend on these methodological factors [19,27,31]. With regard to data comparability, the use of identical protocols for library preparation or RT-qPCR setup is crucial for valid and reproducible comparison of RNA levels between different setups or library preparations. Nevertheless, external natural or artificial sequence-based spike in RNA controls can be useful to evaluate the sensitivity, accuracy, and comparability of RNA quantification experiments [32,33].

In parallel, the introduction of reference materials, laboratory automation, and standardized instrumentation in the entire biomarker development process is helping to minimize operator bias and improve overall reproducibility. Automated platforms and microfluidics for EV purification, RNA extraction, and RT/qPCR/dPCR setups are increasingly available, enabling pre-optimized and standardized high-throughput analyses, and therefore reduced manual variability [19,34].

**Figure 1:**
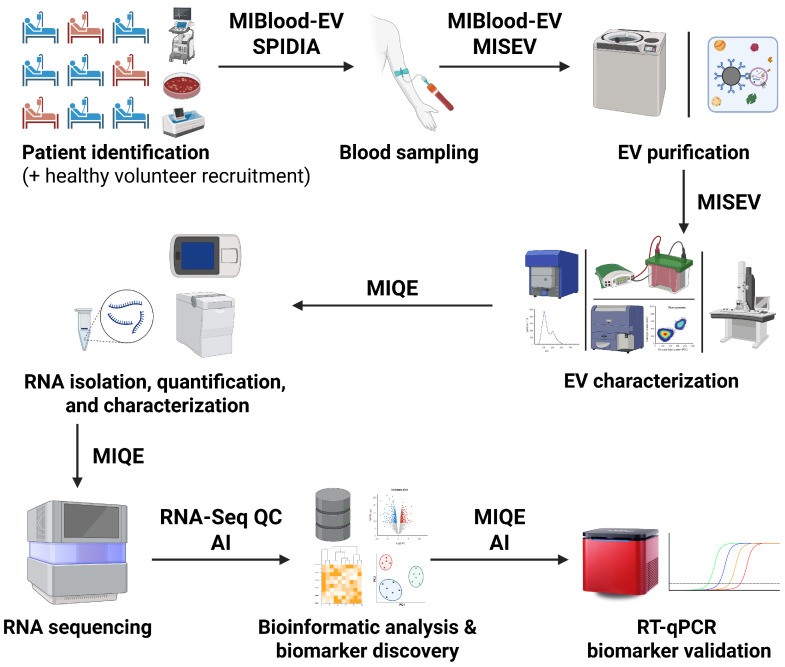
Development and quantification workflow for EV-associated RNA biomarker signatures in liquid biopsy.

These technological advancements, when paired with standardized protocols and continuously updated guidelines, like MIQE, MISEV, SPIDIA, or MIBlood-EV, and implemented wise in the diagnostic workflow (Figure 1) offer a powerful route to more consistent and valid molecular diagnostics and clinically translatable results in liquid biopsy [35,36].

On the other hand, MIQE and MISEV each aim to improve rigor and reproducibility but face recurring limitations, implementation barriers, and controversy in how strictly they should be applied in the daily laboratory practice. In the qPCR field, there is a strong discussion about the MIQE burden versus feasibility. Can we fulfill the long list of all essential and desirable items mentioned in the guideline checklist? Is it too time-consuming and resource-intensive for smaller research laboratories? Some clinical diagnostic units consider all these points on the MIQE checklist as unrealistic for routine work, so they selectively implement only single aspects. Furthermore, there are controversies in interpretation in the scientific community. Some authors might see MIQE as too rigid as the ‘gold standard’ or overarching ‘laws of qPCR’, arguing that strict checklists might be used dogmatically by reviewers or editors, while others criticize that journals do not enforce them strongly enough to change publication practice and the quality of the ‘material & methods’ chapter [37].

Comparable methodological limitations and controversies were reported for MISEV. The first MISEV publication [12] formulated strict requirements and was therefore criticized as over-prescriptive, and likewise drives the field into a burden versus feasibility discussion. The later MISEV versions reframed these large checklists more in an informative style and formulated recommendations, which reduced pushbacks, but also weakens enforceability [13]. Particularly within the EV community, methodological limitations and implementation barriers exist due to the broad heterogeneity of the vesicles, the numerous EV biomarker types, and the method dependency of the results. EV populations, isolation technologies, and analytical platforms are highly diverse, so a single checklist cannot cover all contexts [38,39,40]. Researchers are still debating what constitutes the ‘minimal characteristics’ and how many features, biomarkers, or functional tests are truly needed. The real implementation gap in many EV studies still falls short of MISEV recommendations, since limited markers, poor reporting of biofluid handling, and inadequate controls are used. The strict compliance in clinical settings or with high sample throughput is difficult due to limited EV sample volume, immense characterization costs, and instrumental limitations [16,38]. Several recent articles explicitly focus on reproducibility in EV research and note that even when MISEV and MIQE checklists are strictly followed, substantial between-study variability remains, especially for EV-derived RNA and protein biomarkers. These papers emphasize that guideline adherence mainly improves transparency and comparability, but does not guarantee that a given biomarker will validate in independent cohorts or with alternative isolation and detection workflows [16,39,40].

Despite the disadvantages, it is important to emphasize that all the revised guidelines should not be seen as optional recommendations, but more like a chance and as ‘the’ essential pillars of scientific rigor and standardization in order to achieve better and more meaningful molecular biological results and to promote the integrity of research. Adhering to them can significantly reduce technical or man-made variability and increase the biological and clinical relevance of EV-associated CNA biomarker signatures [30,41,42,43].

## 4. Conclusions

The field of CNA biomarkers, especially those derived from EVs, is rapidly advancing, offering immense promise for ‘non-invasive’ disease diagnostics and monitoring. The overarching goal is the development of robust, sensitive, and early-appearing biomarker signatures with a novel diagnostic value, for different diseases or cancer types, showing high validity and reproducibility [6,7]. I personally believe that the scientific community should recognize this new potential and put it as soon as possible into the research laboratory and, later on, into clinical practice. However, this requires overcoming persistent challenges related to methodological variability and a lack of standardization in the biomarker discovery process [42]. My personal view is that the recent updates and strict revisions of the international guidelines, like MIQE and MISEV, represent a major step forward in promoting methodological transparency, analytical reproducibility, data integrity, and reliability of developed biomarker signatures in liquid biopsy molecular diagnostics. The strict implementation of the MIQE guidelines ensures that nucleic acid quantification is scientifically sound and interpretable, while MISEV standardizes the isolation and characterization of EVs, the central carriers of various molecular biomarkers. We will have a major advantage by integrating these two frameworks into our daily molecular research and in future into clinical practice. Finally, we want to reduce unwanted background variability, enhance the validity of CNA biomarker signatures, and therefore get closer to truly quantitative, reliable biological results [13,43].

Nevertheless, we must also point out the disadvantages, which initially lead to increased effort in implementing the new guidelines into the molecular workflow. This is reflected in more extensive instrumentation, expensive advanced software, more detailed but precise documentation, as well as higher costs for consumables or kits due to established standard materials and more technical replicates. However, it seems evident that these additional investments and efforts will pay off in the long run through better and more valid molecular results that are consistent with real biological measurements. These advances not only benefit academic and clinical research but also accelerate the translation of molecular diagnostics into precision medicine. As automation increases, sensitive microfluidics and bioinformatics tools mature, adherence to these standardized approaches will become even more crucial. I also believe that the increasing use of artificial intelligence in workflow optimization, data correction due to outliers, or data adjustment in the case of missing data points will shape the future and lead to more consistent results [44,45].

My vision and advice to come closer to valid biomarker signatures and for solving these mentioned problems is that the way forward lies in smart, harmonized, transparent workflows—***from liquid biopsy to CNA biomarker signature***—that ensure every reported EV-associated nucleic acid biomarker is based on reproducible science. With continued commitment of the scientific community to these developed guidelines, based on standardized workflows and established quantitative principles, the molecular diagnostic field is poised to deliver on the full potential of EV-associated CNA in liquid biopsy.

## Data Availability

No new data were created or analyzed in this study. Data sharing is not applicable to this article.

## Abbreviations

The following abbreviations are used in this manuscript:

cfRNA	circulating free RNA
CNA	circulating nucleic acids
ctDNA	circulating tumor DNA
dPCR	digital PCR
CEN/TS	European Committee for Technical Specification
EV	extracellular vesicles
HDL	high density lipoprotein
lncRNA	long non-coding RNA
LDL	low density lipoprotein
miRNA	microRNA
mRNA	messenger RNA
isomiRs	microRNA isoforms
MIBlood-EV	Minimal Information for Blood EV research
MIQE	Minimum Information for Publication of Quantitative Real-Time PCR Experiments
MISEV	Minimal Information for Studies of Extracellular Vesicles
NGS	next-generation sequencing
PCR	polymerase chain reaction
qPCR	quantitative polymerase chain reaction
RT	reverse transcription
SPIDIA	standardisation and improvement of generic Pre-analytical tools and procedures for In-vitro DIAgnostics
TEV	tumor-derived extracellular vesicles
VLDL	very low density lipoprotein
